# The *PTPN22 *C1858T gene variant is associated with proinsulin in new-onset type 1 diabetes

**DOI:** 10.1186/1471-2350-12-41

**Published:** 2011-03-23

**Authors:** Lotte B Nielsen, Sven Pörksen, Marie Louise M Andersen, Siri Fredheim, Jannet Svensson, Philip Hougaard, Maurizio Vanelli, Jan Åman, Henrik B Mortensen, Lars Hansen

**Affiliations:** 1 Department of Paediatrics, Glostrup University Hospital, Denmark; 2 Department of Statistics, University of Southern Denmark; 3 Department of Paediatrics, University of Parma, Italy; 4 Department of Paediatrics, Örebro University Hospital, Sweden

## Abstract

**Background:**

The protein tyrosine phosphatase nonreceptor type 2 (*PTPN22*) has been established as a type 1 diabetes susceptibility gene. A recent study found the C1858T variant of this gene to be associated with lower residual fasting C-peptide levels and poorer glycemic control in patients with type 1 diabetes. We investigated the association of the C1858T variant with residual beta-cell function (as assessed by stimulated C-peptide, proinsulin and insulin dose-adjusted HbA_1c_), glycemic control, daily insulin requirements, diabetic ketoacidosis (DKA) and diabetes-related autoantibodies (IA-2A, GADA, ICA, ZnT8Ab) in children during the first year after diagnosis of type 1 diabetes.

**Methods:**

The C1858T variant was genotyped in an international cohort of children (n = 257 patients) with newly diagnosed type 1 diabetes during 12 months after onset. We investigated the association of this variant with liquid-meal stimulated beta-cell function (proinsulin and C-peptide) and antibody status 1, 6 and 12 months after onset. In addition HbA_1c _and daily insulin requirements were determined 1, 3, 6, 9 and 12 months after diagnosis. DKA was defined at disease onset.

**Results:**

A repeated measurement model of all time points showed the stimulated proinsulin level is significantly higher (22%, p = 0.03) for the T allele carriers the first year after onset. We also found a significant positive association between proinsulin and IA levels (est.: 1.12, p = 0.002), which did not influence the association between *PTPN22 *and proinsulin (est.: 1.28, p = 0.03).

**Conclusions:**

The T allele of the C1858T variant is positively associated with proinsulin levels during the first 12 months in newly diagnosed type 1 diabetes children.

## Background

Type 1 diabetes is a T-cell mediated autoimmune disease leading to beta-cell destruction and loss of insulin secretion resulting in severe hyperglycemia. Type 1 diabetes results from a complex interaction between environmental and genetic factors. Several genes have been identified as causative in the development of type 1 diabetes [1,2] and some of these genes as well as other genes are shown to exert an impact on the disease progression from onset in newly diagnosed type 1 diabetes children [3-6]. In a number of studies, the non-synonymous variant, C1858T, of the *PTPN22 *gene has been associated with development of type 1 diabetes as well as other autoimmune diseases [7-11]. Recently, this *PTPN22 *susceptibility variant was found to be significantly associated to lower fasting C-peptide levels, poorer glycemic control in recent onset type 1 diabetes subjects [6] and to higher GADA in type 1 diabetes patients with long disease duration [12]. The objective of the current longitudinal investigation was therefore to evaluate the impact of *PTPN22 *on disease progression as assessed by liquid meal-stimulated C-peptide and proinsulin, HbA_1c_, daily insulin dose, insulin dose-adjusted HbA_1c _(IDAA1C) [13], antibodies to the protein tyrosine phosphatase related IA-2 molecule (IA-2A), islet cell antibodies (ICA), insulin antibodies (IA), glutamic acid decarboxylase antibodies (GADA) and zinc transporter-8 antibodies (ZnT8Ab) in the Hvidoere Study Group on Childhood Diabetes (HSG) remission phase cohort [14].

## Methods

The study population representing 15 countries in Europe and Japan was collected through HSG and is described in Mortensen et al 2009 [14]. The cohort included 126 girls and 131 boys, 84% of the patients were white Caucasian, and age at clinical diagnosis was 9.1 ± 3.7 years (mean ± SEM), BMI 16.5 ± 3.2 kg/m^2^, and HbA_1c _11.2 ± 2.1% at the time of diagnosis. DKA (HCO_3 _≤ 15 mmol/l and/or pH ≤ 7.30) was present in 20.7% of the cases at the time of diagnosis.

Exclusion criteria were: suspected non-type 1 diabetes (type 2 diabetes, maturity-onset diabetes of the young (MODY) or secondary diabetes), decline of enrolment into the study by patients or parents, and patients initially treated outside of the centres for more than 5 days. There were no significant differences with respect to gender distribution, age, anthropometric data, HbA_1c _at diagnosis, ethnicity or family history of diabetes between patients included and patients not included into the study (data not shown). The diagnosis of type 1 diabetes was according to the World Health Organization criteria. The study was performed according to the criteria of the Helsinki II Declaration and was approved by the local ethic committee in each centre. All patients, their parents or guardians gave informed consent.

In order to estimate the residual beta-cell function (C-peptide and proinsulin) a liquid-meal Boost™-test (6 ml/kg (max: 360 ml, Mead Johnson, Evansville, IN, USA; 237 ml = 8 FL OZ contains 33 g carbohydrate, 15 g protein and 6 g fat, a total of 240 kcal)) was carried out at 1, 6 and 12 months (± 1 week) after diagnosis in all 257 children with newly diagnosed type 1 diabetes. HbA_1c_, IDAA_1c_, insulin regimen, HLA typing, antibodies (except ZnT8Ab) and liquid-meal stimulated C-peptide levels were analyzed centrally [14]. Liquid-meal stimulated proinsulin was analysed by a sandwich ELISA assay using two monoclonal antibodies. The assay detects total proinsulin as well as the four metabolites: split(32-33), des(31-329, split(65-66) and des(64-65)-proinsulin. The detection limit is 0.3 pmol/l and the interassay coefficients of variation are <8.7%. This assay has no cross reactivity with insulin, C-peptide, IGF-I and IGF-II. ZnT8Ab measurements were done as described in [15]. C1858T genotyping (rs2476601) was done using an in-house KASPar system at KBioscience, UK.

## Statistical Analyses

Stimulated C-peptide (logarithmic) and stimulated proinsulin (logarithmic) at 1, 6, 12 months after diagnosis and HbA_1c_, daily insulin dose and IDAA1C at 1, 3, 6, 9, 12 months after diagnosis were analyzed as dependent variables in two separate multiple regression repeated measurements models with unstructured variance with gender, age, genotype and IA as explanatory factors. The assumption of constant effect of genotype was checked by first allowing for interaction between genotype and disease duration. Results are given as the estimated factor between the T allele carriers (CT and TT) and the CC genotype. Autoantibodies were examined by the non-parametric Wilcoxon test for single time points. The relationship between IA and proinsulin was tested by multiple regression analysis at each time point with IA (logarithmic), age, gender and stimulated C-peptide (logarithmic) as explanatory factors. There were no statistical differences between the variables according to genotype groups whether the statistical analyses were performed with or without the four Japanese patients. P values below 0.05 were considered statistically significant.

## Results

The genotype distribution of the *PTPN22 *C1858T variant among the 257 patients from the HSG [186 CC (73%), 65 CT (25%), 6 TT (2%)] was in Hardy-Weinberg equilibrium. The clinical characteristics according to *PTPN22 *genotypes are presented in Table 1. There were no significant genotype differences in clinical and demographic data at disease onset. Excluding the Japanese patients from the following analyses did not change the results.

**Table 1:** Clinical characteristics at onset in type 1 diabetes subjects according to *PTPN22 *C1858T genotypes

The HSG cohort	C1858T genotype		
	**CC**	**CT**	**TT**
Sex (male/female)	90/96	33/32	3/3
Age (yrs)	9.3 ± 0.3	8.8 ± 0.5	6.7 ± 1.1
Presences of DKA (+/-) (%)	22/78	13/87	17/83
BMI (kg/m2)	17.8 ± 0.2	18.0 ± 0.4	16.3 ± 0.6
HLA risk genes: (Caucasian/Japanese = 253/4)			
Low (%)	52/0.8 *	46	17
Moderate (%)	11/0.4 *	14	17
High (%)	37/0.4 *	40	67

Data are mean values ± SEM. HLA *DRB1 *high risk genotypes: *DR 03/04 *and *DR 04/04*; HLA *DRB1 *moderate risk genotypes: *DR 03/03 *and *DR04/08*; all other HLA *DRB1 *genotypes were considered low risk. The HLA *DRB1 *genotypes for the 4 Japanese patients were: *DR 01/09, DR 04/08, DR 09/09 *and *DR 04/09*. These were classified as HLA risk: low, low, moderate and high, respectively* [20].

**Figure 1:**
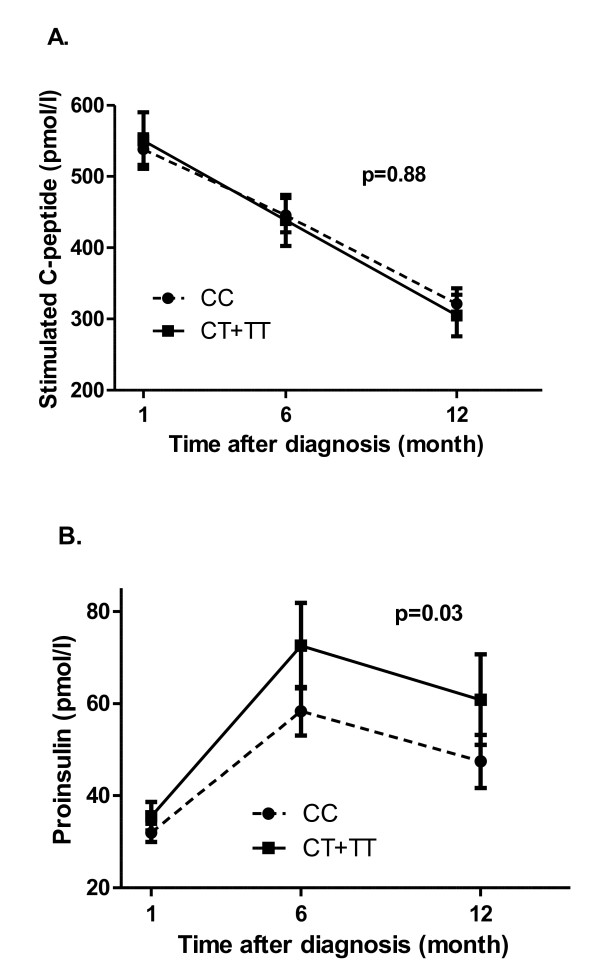
**Stimulated C-peptide and proinsulin over time according to *PTPN22 *genotypes**. The association of stimulated C-peptide (A.) and proinsulin (B.) level 1, 6 and 12 months after disease onset by *PTPN22 *genotype (homozygous+heterozygous (CT+TT) (n = 71) carriers of the variant versus wildtype (CC) (n = 186)). Mean values (pmol/l) ± SEM.

When analyzing the effect of the *PTPN22 *gene variant on the residual beta-cell function in a repeated measurement model (including 1, 6 and 12 months) we did not find an effect on the stimulated C-peptide levels the first year after onset (est.: 1.018, p = 0.88) (Figure 1A). However, when analyzing the relation between proinsulin and carriers of the C1858T variant we find CT and TT carriers had significantly higher proinsulin (30%) levels over the 12 month period compared to the CC genotype group (est.: 1.30, p = 0.03) (Figure 1B). When adjusting for the IA levels in this analysis, we found a significant association between proinsulin and the IA levels (est.: 1.12, p = 0.002), which, however, did not influence the association between the *PTPN22 *gene variant and proinsulin (est.: 1.28, p = 0.03). Furthermore, the CT and TT genotype carriers had a borderline significant higher proinsulin/C-peptide ratio in comparison with the CC genotype carriers (est.: 1.25, p = 0.05).

There were no significant differences in the IDAA1C levels among the *PTPN22 *carriers, an HbA1c and insulin weighted indirect measure of the residual beta-cell function [13]. Nor did we find any differences between carriers of the *PTPN22 *variant and the glycemic control (as assessed by HbA_1c_) or daily insulin dose.

**Figure 2:**
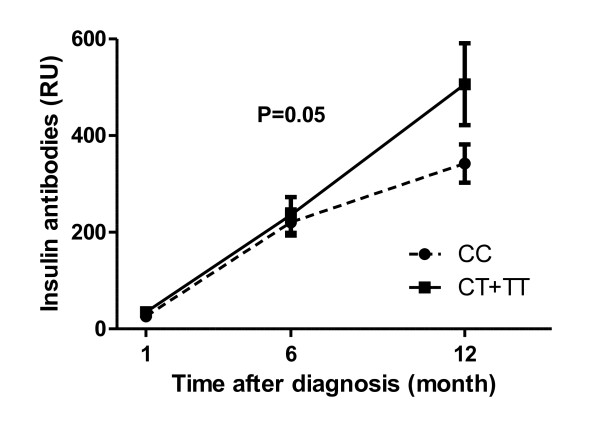
**Insulin antibodies over time according to *PTPN22 *genotypes**. Distribution of insulin antibodies (IA) by *PTPN22 *genotype ((CT+TT) (n = 71) carriers of the variant versus wildtype (CC) (n = 186)) 12 months after disease onset. Mean values (pmol/l) ± SEM.

Finally a significant association was observed between carriers of the CT and TT genotype groups and high IA levels at 12 months after onset (est.: 1.56, p = 0.05) (Figure 2), while there were no significant difference in the IA levels at 1 and 6 months. The prevalence of ZnT8Ab was 68% 1 month after disease diagnosis (time point nearest to disease onset) for either ZnT8RAb and/or ZnT8WAb. There was no association between the *PTPN22 *variant and the other diabetes-related autoantibodies (ICA, IA-2A, GADA and ZnT8Ab), neither close to onset or later during disease progression.

## Discussion

The *PTPN22 *gene is shown in numerous studies to be associated with the development of type 1 diabetes and other autoimmune diseases. The gene encodes a lymphoid tyrosine phosphatase (LYP) which by dephosphorylation of Src family kinases negatively regulates T cell receptor (TCR) signalling. The current working hypothesis suggest that the risk carrying allele, T1858, suppresses TCR signalling more efficiently during thymic development resulting in survival of autoreactive T-cells [16].

Bottini N and co-workers [16] suggested the use of *PTPN22 *SNPs as a prognostic factor for disease severity and variability in autoimmune diseases, but also requested further studies taking this point under investigation. One such study was recently reported in 120 new onset type 1 diabetes patients (from 5 to 36 years) [6]. The authors found an association between the T allele of the C1858T variant (using a dominant model) and low fasting C-peptide (as a surrogate marker of residual beta-cell mass) and poorer glycemic control (HbA_1c_) from onset and during 12 months follow-up. The relationship between carriers of the T allele and proinsulin was not investigated. The present study, which was conducted in 257 children (from 2 to 16 years) (Table 1) with new onset type 1 diabetes, did not find a direct correlation between stimulated C-peptide and the *PTPN22 *variant (Figure 1A), but suggests an association between carriers of the T allele and high proinsulin throughout the first year after disease onset (Figure 1B). In accordance with this, an increased proinsulin/C-peptide ratio was evident in the T allele carriers. Previously, elevated proinsulin and proinsulin/C-peptide ratio was found in non-diabetic first-degree relatives positive for islets autoantibodies to identify individuals with increased risk of developing type 1 diabetes within 5 years [17]. Furthermore, elevated proinsulin levels were found to reflect an impaired beta-cell function in type 2 diabetes patients [18]. Thus, the increased proinsulin level and proinsulin/C-peptide ratio might either be due to impaired proinsulin processing or increased secretory demand on the beta-cells resulting from either autoimmunity or hyperglycemia induced residual beta-cell stress.

Circulating IA's are known to change the metabolic clearance rate of proinsulin through proinsulin binding to IgG [19], correspondingly we found a significant positive association between proinsulin and IA levels, irrespective of genotypes (Table 2). This relation did not affect the association between proinsulin and the *PTPN22 *variant in the statistical analyses. There was a tendency of higher IA levels among the T allele carriers 12 months after onset (Figure 2), pointing towards a genuine association between IA and the *PTPN22 *variant, independent of proinsulin. A plausible explanation for these findings could be that *PTPN22 *1858T is involved in two initially independent processes: first, the severity of autoimmune destruction of beta-cells evidenced by its association with higher proinsulin, this would be in accordance with the data reported by Petrone et al. [6] and second, that *PTPN22 *1858T is also involved in the antibody response to exogenous insulin treatment. In our case these two processes converge because of the effect that insulin antibodies have on the metabolic clearance rate of proinsulin. Thus, carriers of the *PTPN22 *1858T allele seem to have both a higher proinsulin and a higher IA level during disease progression despite they are treated with comparable insulin dose as CC carriers.

**Table 2:** The relationship between IA and proinsulin.

	Doubling the level of IA		
	**1 mth**	**6 mths**	**12 mths**
Proinsulin	7%	11%	16%
	P = 0.002	P = 0.007	P < 0.00001

IA levels were associated with proinsulin independent of *PTPN22 *genotypes as a 100% increase of the IA corresponded to a significant increase in proinsulin release the first 12 months after disease onset.

The discrepancies in significant/non-significant findings on C-peptide levels between this study and the study mentioned above [6] may relate to the use of fasting contra stimulated C-peptide. In the fasting state the differences between the genotype groups and C-peptide are apparent while during liquid-meal stimulated conditions the beta-cells are probably capable of compensating the hyperglycemia but with a rise in proinsulin indicating the stressed residual beta-cell function. Furthermore, the differences in the mean age between the patients from the two studies (14.9 yrs vs 9.1 yrs, Petrone et al vs. our study, respectively) might also partly explain the different results. Younger children have less residual beta-cell function as assessed by stimulated C-peptide the first year after diagnosis compared to older age groups [13]. GADA were not related to the *PTPN22 *variant, supporting previous findings that impact of this gene on the presence of GADA may only be observed in patients with long disease duration (> 10 years) [12].

## Conclusion

Our results align with previous observations that *PTPN22 *gene variant may be associated with changes in residual beta-cell function and disease pathogenesis during the first year after onset of type 1 diabetes.

## List of abbreviations

IDAA1C: Insulin dose-adjusted HbA_1c_; HSG: Hvidoere Study Group on Childhood Diabetes; DR: Danish Remission Phase Study; IA-2A: protein tyrosine phosphatase related IA-2 molecule; GADA: glutamic acid decarboxylase antibodies; IAA: insulin antibodies; ICA: islet cell antibodies, ZnT8Ab: zinc transporter 8 antibodies; SNP: Single nucleotide polymorphism

## Competing interests

The authors declare that they have no competing interests.

## Authors' contributions

LBN designed the study, contributed to data analysis and wrote the manuscript, SP contributed to data analysis and discussion, MLMA contributed to data analysis, SF contributed to data management, JS contributed to manuscript preparation, PH did the statistical analysis, MV provided the sample and clinical data of study participants, JÅ provided the sample and clinical data of study participants,, HBM contributed to data discussion and manuscript preparation, LH contributed to data analysis, discussion and manuscript preparation. All authors read and approved the manuscript.

## Pre-publication history

The pre-publication history for this paper can be accessed here:

http://www.biomedcentral.com/1471-2350/12/41/prepub
